# Utilizing Robust Design to Optimize Composite Bioadhesive for Promoting Dermal Wound Repair

**DOI:** 10.3390/polym15081905

**Published:** 2023-04-15

**Authors:** Rattapol Pinnaratip, Zhongtian Zhang, Ariana Smies, Pegah Kord Forooshani, Xiaoqing Tang, Rupak M Rajachar, Bruce P. Lee

**Affiliations:** 1 Department of Biomedical Engineering, Michigan Technological University, Houghton, MI 49931, USA; rpinnara@mtu.edu (R.P.);; 2 Department of Biological Sciences, Life Science and Technology Institute, Michigan Technological University, Houghton, MI 49931, USA; 3 Marine Ecology and Telemetry Research (MarEcoTel), Seabeck, WA 98380, USA

**Keywords:** catechol-based adhesive, silica particle, dermal wound repair, hydrogen peroxide, robust design

## Abstract

Catechol-modified bioadhesives generate hydrogen peroxide (H_2_O_2_) during the process of curing. A robust design experiment was utilized to tune the H_2_O_2_ release profile and adhesive performance of a catechol-modified polyethylene glycol (PEG) containing silica particles (SiP). An L_9_ orthogonal array was used to determine the relative contributions of four factors (the PEG architecture, PEG concentration, phosphate-buffered saline (PBS) concentration, and SiP concentration) at three factor levels to the performance of the composite adhesive. The PEG architecture and SiP wt% contributed the most to the variation in the results associated with the H_2_O_2_ release profile, as both factors affected the crosslinking of the adhesive matrix and SiP actively degraded the H_2_O_2_. The predicted values from this robust design experiment were used to select the adhesive formulations that released 40–80 µM of H_2_O_2_ and evaluate their ability to promote wound healing in a full-thickness murine dermal wound model. The treatment with the composite adhesive drastically increased the rate of the wound healing when compared to the untreated controls, while minimizing the epidermal hyperplasia. The release of H_2_O_2_ from the catechol and soluble silica from the SiP contributed to the recruitment of keratinocytes to the wound site and effectively promoted the wound healing.

## 1. Introduction

Rapid dermal healing requires a balance of redox control [1,2]. During the early phases of the wound healing process, macrophages and neutrophils are attracted to the wound site and release reactive oxygen species (ROS), such as hydrogen peroxide (H_2_O_2_), at concentrations within the micromolar range. H_2_O_2_ induces vascular endothelial growth factor (VEGF) expression in keratinocytes, [3] which stimulates angiogenesis in wounds [4]. ROS are also necessary in the differentiation of M2 macrophages, [5] which promotes tissue regeneration and anti-inflammatory responses [6,7]. The application of ROS to chronic ulcers (e.g., the direct application of H_2_O_2_, hyperbaric treatment to enhance ROS concentration, and the application of honey, etc.) has been found to accelerate healing [2,8]. Additionally, ROS are a natural disinfectant and can prevent bacterial infection [9]. However, high levels of ROS can severely damage healthy tissues, which can lead to the formation of chronic wounds and tumor initiation [10,11]. Biomaterials supplemented with antioxidants have been found to accelerate wound healing, reduce chronic inflammation, and increase biocompatibility [12,13]. However, the complete removal of ROS delays wound healing [2]. As such, tuning the ROS release from a bioadhesive is necessary to promoting rapid dermal wound healing.

Catechol-modified bioadhesives have been widely adopted as biomaterials for various applications, ranging from soft tissue repair to tissue engineering [14,15,16,17]. Catechol mimics the crosslinking and interfacial bonding chemistry of the amino acid, 3,4-dihydroxyphenyl alanine (DOPA), which is the main adhesive molecule found in mussel adhesive proteins. To activate a catechol-based adhesive for curing and adhesion, an oxidant such as tyrosinase or sodium periodate (NaIO_4_) is typically added to initiate the catechol’s oxidation and crosslinking [18,19,20]. During the process of catechol oxidation, micromolar amounts of H_2_O_2_ are generated as by-products [21,22]. To modulate the amount of released H_2_O_2_, we have previously incorporated highly porous and micron-sized silica particles (SiP) into a catechol-functionalized branched polyethylene glycol (PEG) [23]. The silanol (SiOH) surface of these particles absorbs H_2_O_2_ through complexation with water molecules and facilitates the decomposition of H_2_O_2_ to water and oxygen [24]. Additionally, SiP provides cellular binding sites to enhance the bioactivity of the bioinert, synthetic PEG-based adhesive [23]. While these previous studies have demonstrated the ability of SiP to control the H_2_O_2_ production of catechol and improve the biocompatibility of catechol-based bioadhesives [23,25], these materials are not specifically tailored for a given application. Given that the ideal concentrations of H_2_O_2_ needed to promote successful wound healing outcomes are different between tissue types, it is necessary to modulate the release of H_2_O_2_ specifically toward dermal wound healing.

**Scheme 1:**
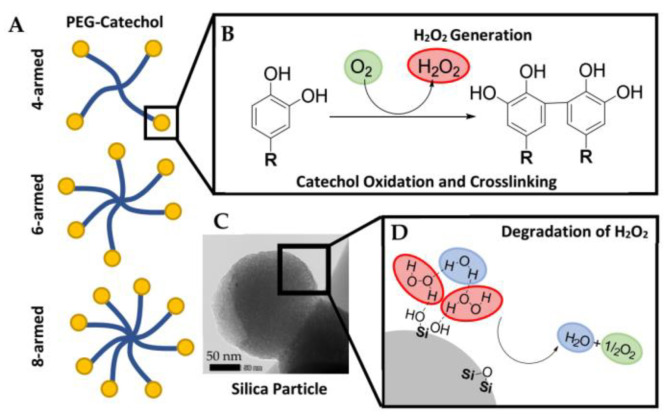
(**A**) Schematic representation of branched PEG–catechol with different number of arms. (**B**) Catechol oxidation and crosslinking. H_2_O_2_ is generated during the process. (**C**) Scanning transmission electron microscopy image of porous SiP. (**D**) Degradation of H_2_O_2_ on the surface of SiP through the complexation with water molecules to form water and oxygen.

In this study, we sought to simultaneously tune the H_2_O_2_ release profile and adhesive performance of a PEG–catechol adhesive incorporated with SiP (Scheme 1) and utilize the composite adhesive for dermal wound repair. However, there are a large number of parameters that may simultaneously affect both the H_2_O_2_ release profile and the functional performance of the adhesive. This yields a multitude of potential adhesive formulations, which can be time consuming to screen. For example, increasing the number of arms in the PEG architecture and PEG concentration effectively increases the crosslinking density of the adhesive, which will enhance the rate of the adhesive curing and adhesive strength [26]. The level of H_2_O_2_ released from a polymer network will depend on the crosslinking density of the matrix, which will trap the generated ROS [22]. Similarly, increasing the SiP concentration in a composite will increase the crosslinking density of the adhesive matrix and enhance both the curing rate and adhesive strength [23]. The concentration of the SiP will also affect the extent of the H_2_O_2_ degradation [25]. Finally, the H_2_O_2_ can acidify the surrounding media [27], which can reduce the rate of the catechol crosslinking and adhesive strength [19]. The concentration of the phosphate-buffered saline (PBS) will be used to minimize the effect of the released H_2_O_2_ while maintaining the buffering capacity of the PBS.

The main objective of this paper is to optimize a catechol-based composite adhesive for dermal wound healing. To this end, the contribution of different parameters to the H_2_O_2_ release profile and adhesive performance, such as the SiP content and adhesive polymer’s architecture and concentration, was evaluated. To screen a large library of adhesive formulations more efficiently and effectively, a robust design experiment was employed. A robust design experiment uses an orthogonal array to make pairwise comparisons between factor levels and permits the investigator to reliably estimate the factor effects with fewer experiments [28,29]. An L_9_ orthogonal array was used to determine the relative contributions of four factors (the PEG architecture, PEG concentration, PBS concentration, and SiP concentration) to the performance of the composite adhesive, as measured by its gelation time, adhesive strength, and the concentration of the H_2_O_2_ generated. The candidate adhesive formulations were selected based on the robust design experiment and their efficacy in promoting dermal wound healing was further evaluated in a full-thickness dermal wound in mice.

## 2. Materials and Methods

### 2.1. Materials

Ethanol (200 proof), tetraethyl orthosilicate (TEOS, 99.8%), PBS (0.137 M NaCl, 0.0027 M KCl, and 0.0119 M phosphates), acetic acid (Glacial), sodium hydroxide, Dulbecco’s modified eagle medium, fetal bovine serum, and Penicillin-Streptomycin were obtained from Fisher Scientific Co. (Pittsburgh, PA, USA). Sodium periodate (NaIO_4_, >99.8%) was purchased from Acros Organics (Fair Lawn, NJ, USA). A Trichrome Stain (Masson) Kit, the histology mounting medium Polyfreeze, Weigert’s iron hematoxylin solution, and Bouin’s solution were purchased from Sigma-Aldrich (St. Louis, MO, USA). 4,6-Diamidino-2-phenylindole (DAPI) was purchased from Invitrogen (Grand Island, NY, USA). Anti-CD68 antibody (ab125212), Anti-cytokeratin 6 antibody (ab24646), goat anti-rabbit IgG H&L (Alexa Fluor 647), and goat anti-rabbit IgG H&L (Alexa Fluor 488; ab150077) were obtained from Abcam (Cambridge, MA, USA). A Vectastain Elite ABC kit (PK 6101) and Vectastain DAB substrate kit for peroxidase (SK 4100) were purchased from Vector Laboratories (Newark, CA, USA). 4-, 6-, and 8-arm PEG (MW = 10, 15, and 20 kDa, respectively) were purchased from JenKem Technology USA, Inc. (Plano, TX, USA) and were used to prepare the PEG terminated with dopamine (Figure S1, Supplementary Materials), following the previously published protocols [23]. Bovine pericardium tissues were purchased from Sierra for Medical Science (Whittier, CA, USA). Ferrous Oxidation Xylenol Orange (FOX) assay (Quantitative Peroxide Assay Kit) was purchased from Thermo Fisher Scientific (Waltham, MA, USA). The ^1^H NMR confirmed the structures of the prepared PEG terminated with dopamine (Figure S2, Supplementary Materials). The dopamine-modified PEG are abbreviated as PEG-D4, PEG-D6, and PEG-D8, where the number corresponds to the number of arms in the PEG architecture. The porous SiPs were prepared using previously published protocols [25]. A scanning transmission electron microscope was used to confirm the porous surfaces of the SiPs (Figure S3, Supplementary Materials).

### 2.2. Robust Design Experiment

An L_9_ orthogonal array was used to determine the contribution of four factors and their relative contributions to the performance of the composite adhesive (Table 1) [28,29,30]. These factors were: (A) the PEG architecture, (B) the PEG precursor concentration, (C) the PBS concentration, and (D) the SiP concentration. Each factor was tested at 3 levels (e.g., A1, A2, and A3 for the PEG architecture, corresponding to 4-arm, 6-arm, and 8-arm, respectively). To determine the effect of the 4 factors, each at 3 levels, the orthogonal array required the testing of nine adhesive formulations (Table 2). The gelation times, lap shear adhesion strengths, and maximum H_2_O_2_ concentrations of these nine adhesive formulations were determined and these experimental values were utilized to determine the % relative variation or the relative contribution of each factor to the measured outcomes. Additionally, these results were further used to predict the performance of 4^3^ or the 81 possible adhesive formulations within this matrix. The predicted values were utilized to select the adhesive formulations that were potentially suitable for the subsequent dermal wound repair model in mice. A detailed data analysis for the robust design experiment is provided in the Supplementary Materials file.

### 2.3. Preparation of the Composite Adhesive

In total, nine adhesive formulations were prepared based on the desired factor and factor levels shown in Table 2. Polymer precursor solutions were prepared by dissolving the corresponding PEG adhesives with the corresponding PBS solutions, according to Table 2. The composite adhesives were prepared by mixing equal volumes of the polymer precursor and NaIO_4_ (11.6 mg/mL in deionized water) solutions [25]. NaIO_4_ was used to oxidize the catechol and initiate the adhesive curing [18,19]. After mixing, the final concentrations of the PEG, PBS, and SiP in the composite adhesive would be reduced by half. As such, it was necessary to double the concentrations of the PEG, PBS, and SiP in the precursor solutions so that their final concentrations were reduced to the desired concentrations shown in Table 2.

### 2.4. Characterization of the Composite Adhesive

The time it took for the composite adhesive to cure was determined by using the vial tilting technique, as described in previous publications [18,19]. Briefly, 100 µL of the polymer precursor and 100 µL of the 11.6 mg/mL of NaIO_4_ dissolved in deionized water were mixed in a vial. The concentrations of the adhesive polymer and the SiP in the polymer precursor solution were prepared based on Table 2. The moment that the adhesive mixture ceased to flow in a tilted vial was recorded as the gelation time.

A lap shear adhesion test was performed using strips of bovine pericardium (2.5 cm × 2.5 cm) as the test substrate, following ASTM standards F2255-05 [31]. In total, 100 μL of the polymer precursor and 100 μL of 11.6 mg/mL of the NaIO_4_ solutions were mixed in a glass vial and quickly added onto a piece of pericardium tissue. A second piece of pericardium tissue was then applied over the adhesive to create an adhesive joint with an overlapping area of 1 cm × 2.5 cm. The adhesive joint was weighted down using a 100 g weight for 15 min and further incubated in the PBS (pH = 7.4) at 37 °C overnight. The dimensions of the overlapped area were measured using a digital caliper for each sample before the adhesion testing. The samples were pulled to failure using an Electroforce^®^ machine (Bose Electroforce Group, Eden Prairie, MN, USA) at a speed of 0.1 mm/s. The lap shear adhesive strength was calculated by dividing the maximum load by the overlapped area of the adhesive joint.

A rheological analysis was performed using a Discovery Hybrid Rheometer (TA Instruments, New Castle, DE, USA), using cure adhesive samples that were cut to a disc shape (diameter = 10 mm, thickness = 5 mm, and n = 3). Amplitude sweep experiments (0.1–100% at 0.1 Hz) were performed using parallel plates at a gap distance that was set to be 87.5% of the thickness of the individual sample, as measured by a digital caliper. A PerkinElmer Spectrum One spectrometer was used to perform a Fourier transform infrared (FTIR) spectroscopy analysis on the freeze-dried adhesive samples.

FOX assay was utilized to quantitatively measure the amount of H_2_O_2_ generated from the composite adhesive [22]. The adhesives were cured in the shape of a disc with a diameter of 10 mm and incubated with 1.5 mL of Dulbecco’s modified eagle medium (Corning Cellgro, Manassas, VA, USA) DMEM with 10% (*v*/*v*) fetal bovine serum (FBS) and 0.5% (*v*/*v*) Penicillin-Streptomycin with phenol red (pH = 7.4) for 6 h at 37 °C. The concentration of the H_2_O_2_ was determined by mixing 20 μL of the hydrogel extract with 200 μL of the FOX reagent, incubating the mixture at room temperature for 20 min, and then analyzing the absorbance of the mixture via a plate reader (SynergyTM HT, BioTek, Santa Clara, CA, USA) at 590 nm.

### 2.5. Full Thickness Dermal Wound Repair Model

The ability of the adhesive to promote wound healing in a full-thickness wound model was examined using the published protocols with minor modifications (Figure S4, Supplementary Materials) [32,33,34]. The protocol (Board Ref# L0270) was approved by the Institutional Animal Care and Use Committee (IACUC) at Michigan Technological University on 12/14/2015. Briefly, 17 healthy female wild-type C57BL/6J mice (#000664, the Jackson Laboratory; age 9–10 weeks, weight 20 g) were anesthetized with isoflurane. The hair of the animals was removed from the potential dorsal wound sites with an electric shear and hair removal cream. The next day, the mice were anesthetized and 2 wounds were created bilaterally on the back of the mice using a 5 mm tissue punch. A medical-grade silicon ring (outer diameter = 10 mm and inner diameter = 6 mm) was fixated around each wound using cyanoacrylate glue and 5-0 nylon sutures. The ring served as a splint to minimize the skin movement and a reduction in the wound size as a result of skin contraction. The wound was either left untreated (control) or was treated with one of four adhesive formulations. The number of repetitions per treatment per time point was three. The adhesive was left undisturbed for 2 min to enable it to solidify before the wound was covered with a non-adhering dressing (Adaptic^®^) and then a breathable adhesive film (Hydrofilm^®^). A larger piece of Hydrofilm^®^ was further utilized to seal the wounds from their surroundings. Buprenorphine (1 mg/kg) was administered for three days to ensure the animal’s comfort. Images of the wound were taken to determine the size of the wound using an Olympus stereo microscope with a video capture module. On days 7 or 14, the mice were euthanized via CO_2_ asphyxiation and the tissues surrounding the wounds were collected for further analyses.

### 2.6. Histological and Immunological Analysis of Dermal Wounds

The harvested tissue samples from the wound site were fixed in Polyfreeze^®^, flash-frozen in liquid nitrogen, and stored for up to 4 weeks at −80 °C. The tissue sections with a thickness of 10 µm were obtained using a cryomicrotome and further mounted onto Histobond^®^ slides. A total of 10 mounted tissue slides, each containing 2 slices of tissues, were produced for each tissue sample. A histological analysis was performed using Masson’s trichrome staining to evaluate the wound morphology, epidermis thickness, and collagen content [23]. Additionally, keratin 6 was used to stain for keratinocyte and to determine the wound maturity, using a previously established protocol with minor modifications [35]. Specifically, toluidine blue was replaced with hematoxylin. The samples were rinsed with tap water, immersed for 1 to 5 min in the hematoxylin solution, and further washed using running tap water until the water became colorless. The samples were then dipped 10 times in an acetic acid solution (2 mL glacial acetic acid in 98 mL deionized water), 10 times in cool tap water, 5 times in a bluing solution (0.3 mL NH_4_OH in 100 mL tap water), and 20 times in tap water. The samples were mounted using a permanent mounting medium, stored at 4 °C overnight, and imaged using an EVOS microscope under polarized light. The overlaid images were processed using the auto stitching module in Adobe Photoshop (version 24.1.1, Adobe, San Jose, CA, USA) and analyzed using the wound healing tool macro in ImageJ [36].

The dermal wounds tissue slides were stained using Anti-CD68 antibody and Alexa Fluor 488 to visualize the CD68 positive macrophage with DAPI, in order to visualize all the cell nuclei to determine the overall population of macrophages at the wound site [23]. The samples were submerged in 100% ethanol for 2 min and washed 3 times in the PBS with Tween 20 (PBST; 5 min each time). A hydrophobic marker was used to draw a circle around each sample. The samples were incubated in 10% goat serum diluted with 1% bovine serum albumin for 60 min, a 1/100 dilution primary anti-CD68 Ab for 12–14 h at 4 °C in a humidified chamber, a 1/200 dilution of the secondary antibody (goat anti-rabbit IgG H&L) for 60 min at room temperature, and DAPI antibody (1/1000 dilution) for 3 min. After each incubation step, the samples were washed using PBST 3 times, for 5 min each time. The samples were mounted using an aqueous mounting solution and imaged immediately after the staining with an Olympus fluorescence microscope.

### 2.7. Statistical Analysis

The statistical analysis was performed using JMP Pro 13 (SAS Institute, NC, USA). A one-way analysis of variance (ANOVA) with a Tukey–Kramer HSD analysis was performed using a *p* value of 0.05.

## 3. Results

### 3.1. Robust Design Experiments

**Figure 1:**
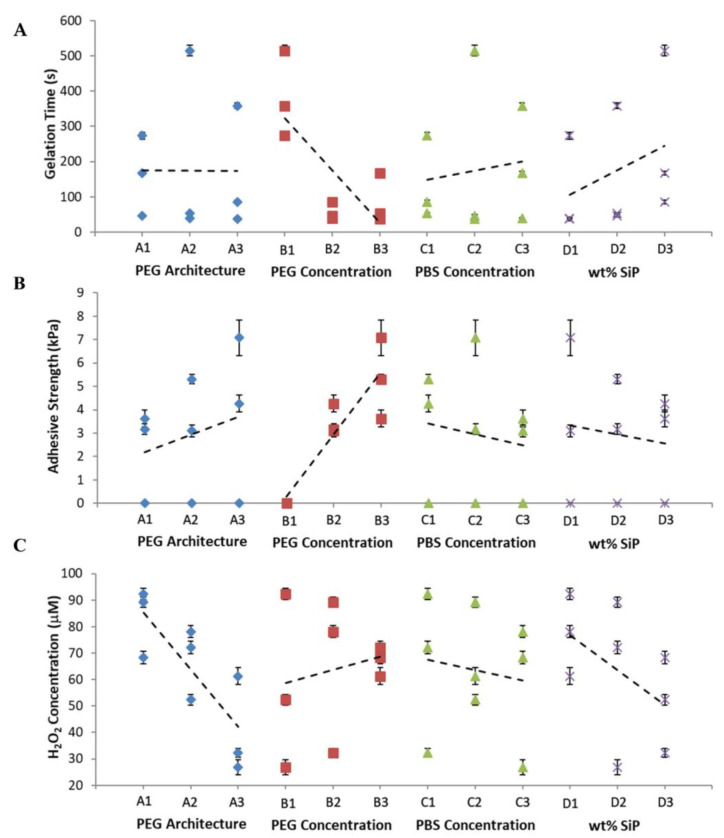
Average gelation time (**A**), adhesive strength (**B**), and H_2_O_2_ concentration (**C**), based on the 9 adhesive formulations from Table 2. Values are plotted as means and standard deviations of experimental values for the corresponding factor levels. The dashed lines are linear trend lines.

An L_9_ orthogonal array was used to determine the relative contributions of four factors (the PEG architecture, PEG concentration, PBS concentration, and SiP concentration) to the performance of the composite adhesive [28,29]. To examine the effect of these four factors, each at three factor levels, the robust design experiments required the testing of nine formulations (Table 2). The gelation times, adhesive strengths, and the amounts of H_2_O_2_ generated for each of the nine formulations were determined (Table S1, Supplementary Materials). The effect of each factor on the performance of the adhesive can be observed in Figure 1. In these plots, the experimental values are plotted against the corresponding factor levels. For example, factor level A1 corresponds to the three data points associated with the four-arm PEG (Table 1), which included data from Formulations 1, 2 and 3 (Table 2). The slope of the linear trend lines indicated how each factor contributed to the performance. For example, the gelation time decreased with an increasing PEG concentration factor level (from B1 to B3). As expected, an increasing polymer concentration increased the rate of curing. However, increasing the SiP wt% had an opposite effect. This is somewhat unexpected, given that the incorporation of SiP as a filler increases the matrix crosslinking density, which should theoretically result in an increased rate of curing [23]. These results may be skewed due to the fact that two of the slowest curing formulations (Formulations 4 and 7) consisted of 75 mg/mL of the PEG, and the low polymer concentration contributed to a slower rate of curing.

For the adhesive property (Figure 1B), the adhesive strength increased with the PEG branching and PEG concentration. Increasing the level of branching increased the crosslinking density and bulk mechanical property of the adhesive, which contributed to a higher lap shear strength [26]. Catechol is responsible for strong interfacial bonding and the catechol concentration increased with an increasing PEG concentration. For the formulations with the lowest PEG concentration of 75 mg/mL (Formulations 1, 4, and 7), the measurable adhesive strength was not demonstrated, which may be due to the low catechol content in these formulations. The H_2_O_2_ concentration decreased with an increase in the PEG branching and SiP wt% (Figure 1C). Increasing the degree of the PEG branching increased the crosslinking density of the adhesive network, which could potentially trap the generated H_2_O_2_ within the adhesive network and limit the amount of H_2_O_2_ released from the adhesive [23]. Similarly, increasing the SiP wt% also increased the crosslinking density of the composite adhesive. Additionally, the porous SiP actively decomposed the H_2_O_2_ [25].

To determine the relative degree to which a particular factor affected the performance of the composite adhesive, the measured results were used to determine the statistical coefficient signal-to-noise ratio (S/N) (Figure S5, Supplementary Materials), which is a logarithmic function of the experimental values (Equation (S1)) [28,29]. As such, the change in the S/N values, as a function of the changing factor levels, mirrored those of the experimental values reported in Figure 1. S/N ratios were further used to determine the % relative variation or the relative contribution of each factor to the measured outcomes: the gelation time, adhesive strength, and H_2_O_2_ concentration (Table 3). The PEG concentration contributed the most to the gelation time and adhesive strength (78.6% and 93.8%, respectively) of the composite adhesive. This indicates that the PEG concentration explains the largest portion of the variation in these two data sets. Similarly, the PEG architecture contributed the most to the measured H_2_O_2_ concentration (65.6%). The SiP wt% was also a minor contributor to the measured gelation time and H_2_O_2_ concentration, with percent relative variation values of 18.2% and 20.7%, respectively. The contribution of the PBS concentration was less than 6% for the three adhesive performances measured, indicating that its contribution was insignificant.

### 3.2. Prediction Based on Robust Design Experiment

**Figure 2:**
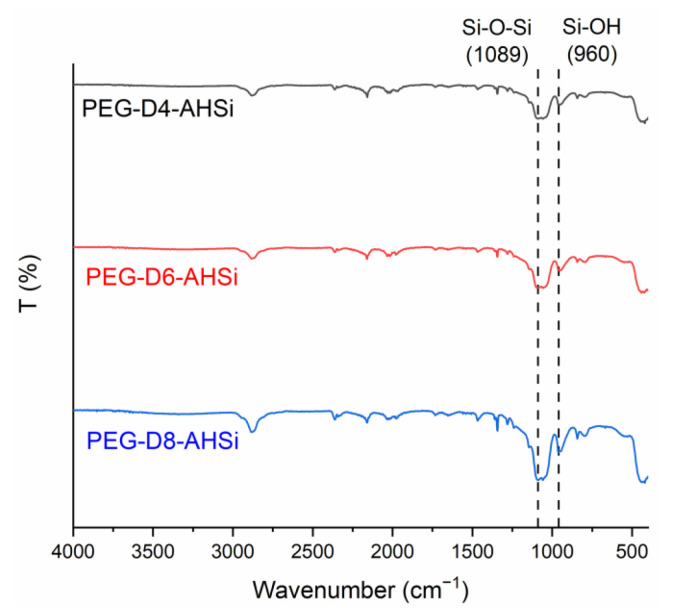
FTIR spectra of the composite adhesives chosen for the dermal wound repair.

**Figure 3:**
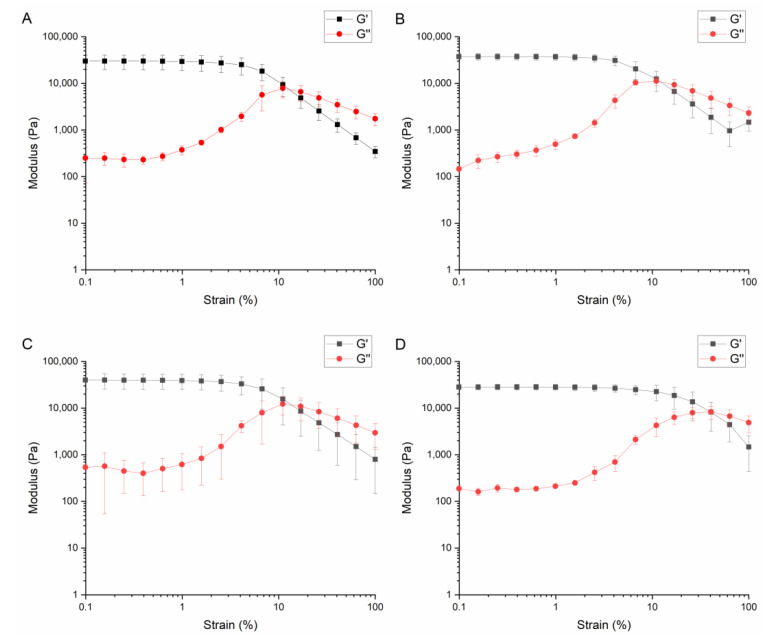
Storage (G′) and loss (G″) modulus of PEG-D6 (**A**), PEG-D6-Si (**B**), PEG-D4-Si (**C**), and PEG-D8-Si (**D**).

The S/N ratios were further utilized to make adhesive performance predictions for the 81 possible formulations (Tables S2–S4) [28]. These predicted values were utilized to select the suitable formulations for the subsequent dermal wound healing model in mice (Tables S5 and S6). All the formulations were selected with the highest PEG concentration of 150 mg/mL, as this factor level yielded the lowest gelation time and the highest adhesive strength. The chosen four formulations also had similar predicted adhesive strength values, ranging from 4.5 to 6.2 kPa. Given that the PBS concentration contributed minimally to the adhesive performance, a 1× PBS concentration was chosen. To evaluate the effect of the H_2_O_2_ concentration on the wound healing, we selected three composite adhesive formulations with increasing branching within the PEG architecture (PEG-D4-Si, PEG-D6-Si, and PEG-D8-Si). The predicted values of the H_2_O_2_ concentration decreased from 86 to 39 µM, with increased branching. Formulations with 10wt% SiP were also chosen, as these formulations released a H_2_O_2_ concentration (50–100 µM) that was in the range that was previously determined to be suitable for wound healing [10,37]. All three formulations contained the same concentrations of PEG and SiP to minimize the effect of the composition on the dermal wound healing. Additionally, PEG-D6 was included as a control and chosen to be compared with PEG-D6-Si, in order to determine the effect of the SiP on the dermal wound repair. FTIR spectra of the composite adhesive exhibited characteristic peaks of Si-OH at 960 cm^−1^ and Si-O-Si at 1089 cm^−1^, which confirmed the presence of SiP within the PEG adhesive (Figure 2). Additionally, oscillatory rheometry confirmed that the adhesives were fully solidified, as the storage modulus (G′) values were higher than those of the loss modulus (G″) values (Figure 3). The G′ values for the different adhesive formulations averaged around 30 kPa.

### 3.3. Validating Results from Robust Design Experiment

**Figure 4:**
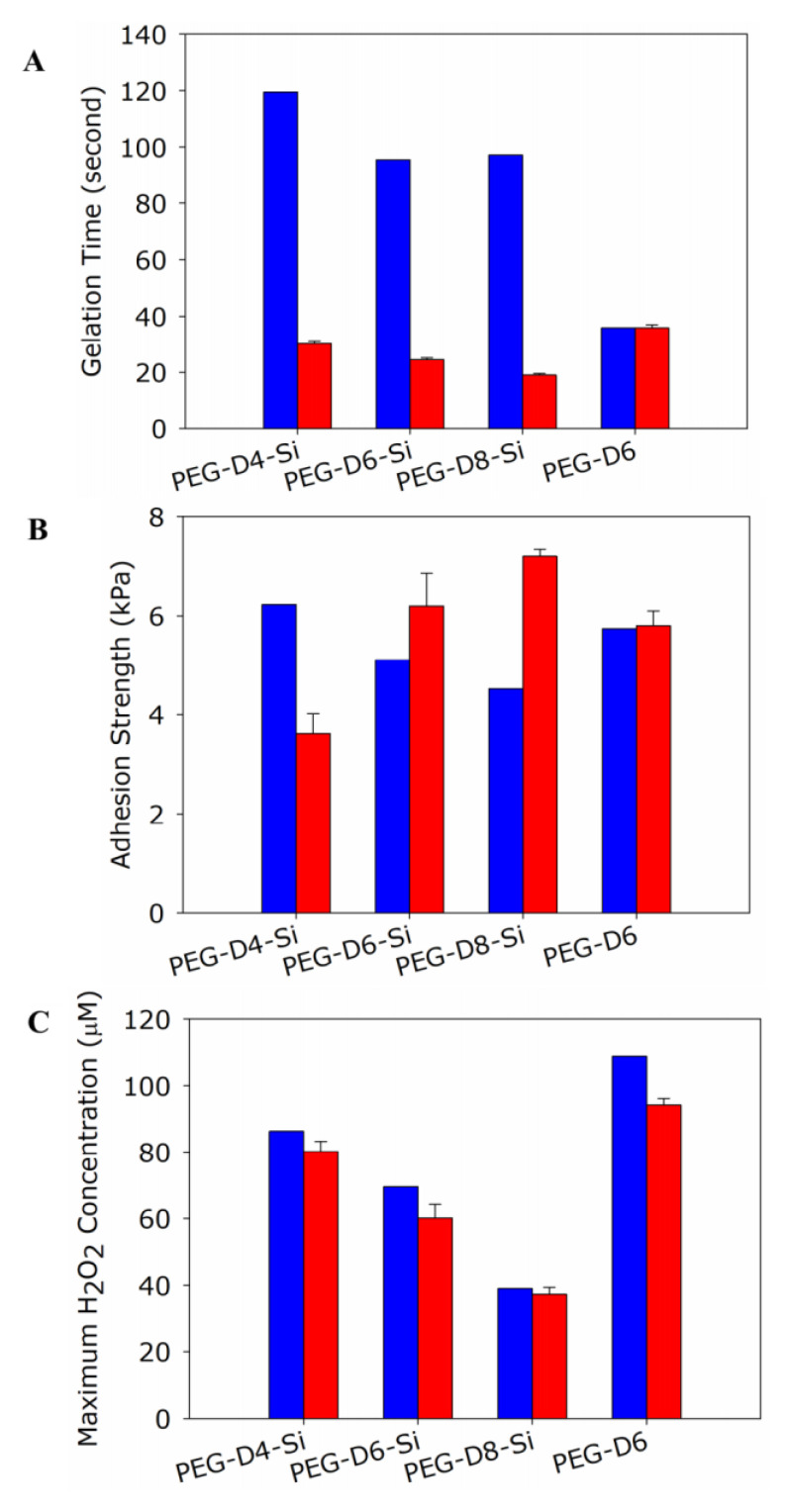
Predicted (■) and experimental (■) results for the gelation time (**A**), adhesion strength (**B**), and H_2_O_2_ concentration (**C**) of the four chosen adhesive formulations. Number of repeats is 3.

The adhesive performances and amounts of H_2_O_2_ generation of the four chosen adhesive formulations were determined and compared with their predicted values (Figure 4). For both the gelation time and adhesive strength, the predicted values matched the experimental values for PEG-D6, which did not contain SiP (a percentage difference of 0.2 and 1.0%, respectively). However, the predictions associated with the SiP-containing formulations were generally poor. The predicted gelation times for the SiP-containing formulations were 4–5 times higher than the experimental values, with a percentage difference of 120–135%. Similarly, the predicted adhesive strength decreased with an increase in the number of PEG arms, which contradicted with the actual experimental data (a percentage difference of 20–45%). When testing the nine adhesive formulations during the robust design experiment, the gelation time increased unexpectedly with an increasing SiP content (Figure 1A), and the adhesive strength decreased unexpectedly with an increasing SiP content (Figure 1B). Both these findings contradict the prior reported findings, where filler concentrations have increased the curing rates and adhesive performances of composite adhesives [23,25].

The observed discrepancy between the predicted and experimental values may be due to the unexpected effect of the formulations that were chosen for the robust design. Formulations 4 and 7 exhibited the highest gelation times and lowest adhesion strengths that were measured, which was mostly likely due to the low PEG concentration (75 mg/mL) in these formulations, rather than a higher concentration of the SiP. The combination of low adhesive polymer concentrations and high SiP concentrations limited the adhesive’s ability to cure and form the strong and cohesive polymer network that is needed to achieve a strong adhesion. However, at a higher adhesive polymer concentration, the SiP contributed to forming a more cohesive network, which enhanced the curing rate and adhesive strength. As such, a higher PEG concentration may be needed for future robust design experiments.

On the other hand, the predicted H_2_O_2_ concentrations matched the experimentally determined values very well, with a percentage difference of 4–14% for the four formulations tested. As expected, the H_2_O_2_ concentration decreased with an increasing number of PEG arms, as a more densely crosslinked network trapped the generated H_2_O_2_ [22]. The PEG-D6-Si also generated less H_2_O_2_ when compared to the PEG-D6, due to the presence of SiP that could decompose the generated ROS. Although the robust design experiment was not accurate in predicting the gelation time and adhesive strength of the composite adhesive, it provided useful guidance in selecting the adhesive formulations based on the amounts of H_2_O_2_ generation.

### 3.4. Dermal Wound Closure

**Figure 5:**
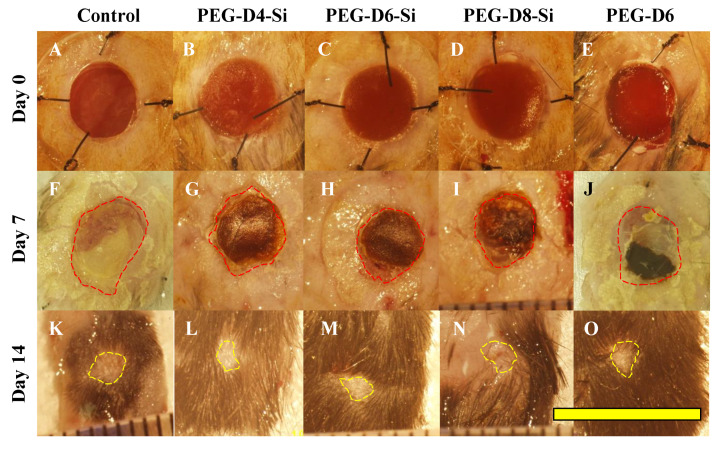
Representative images of the wounds at day 0 (top), 7 (middle), and 14 (bottom) of control (**A**,**F**,**K**), PEG-D4-Si (**B**,**G**,**L**), PEG-D6-Si (**C**,**H**,**M**), PEG-D8-Si (**D**,**I**,**N**), and PEG-D6i (**E**,**J**,**O**). Red and yellow dash lines indicate the wound area. Scale bar = 1 cm.

**Figure 6:**
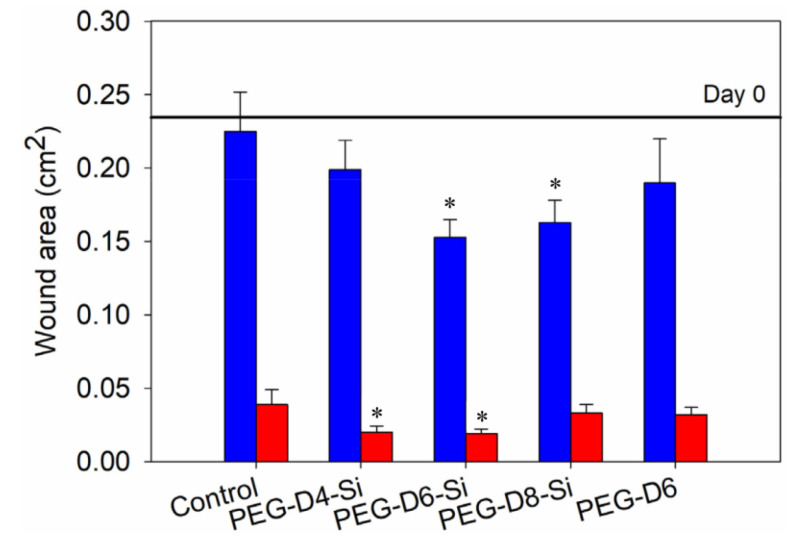
The wound area at days 7 (■) and 14 (■) post-surgery. The horizontal black line at 0.24 cm^2^ represents the initial wound area at day 0. Number of repeats is 3. * *p* < 0.05 when compared to the control for a given time point.

The ability of the composite adhesive to promote dermal wound healing was evaluated using a full-thickness wound healing model in mice. A circular wound with a wound area of 0.24 cm^2^ was created (Figure 5). By day 7, the wound sizes of the adhesive-treated wounds were significantly smaller when compared to the control wound, which was left untreated (Figure 6). Particularly, the wound sizes were reduced by 33–37% for the SiP-containing adhesives, PEG-D6-Si and PEG-D8-Si. By day 14, the wounds treated with the PEG-D4-Si and PEG-D6-Si were found to have the smallest wound sizes, with a reduction in the wound area that was greater than 90%.

**Figure 7:**
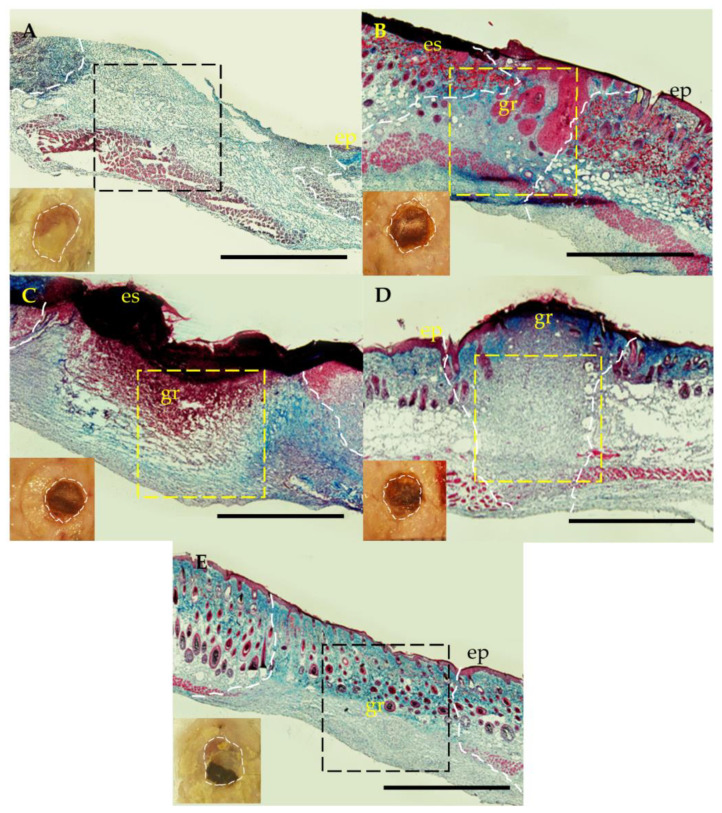
Representative trichrome histological image of the harvested tissue on day 7 after treatment with control (**A**), PEG-D4-Si (**B**), PEG-D6-Si (**C**), PEG-D8-Si (**D**), and PEG-D6 (**E**). Black (**A**,**E**) and yellow (**B**–**D**) dash lines indicate the area used to calculate collagen content. White dash line indicates the wound proximities. es = eschar, gr = granulation tissue, and ep = epidermis. Scale bars are 1 mm.

**Figure 8:**
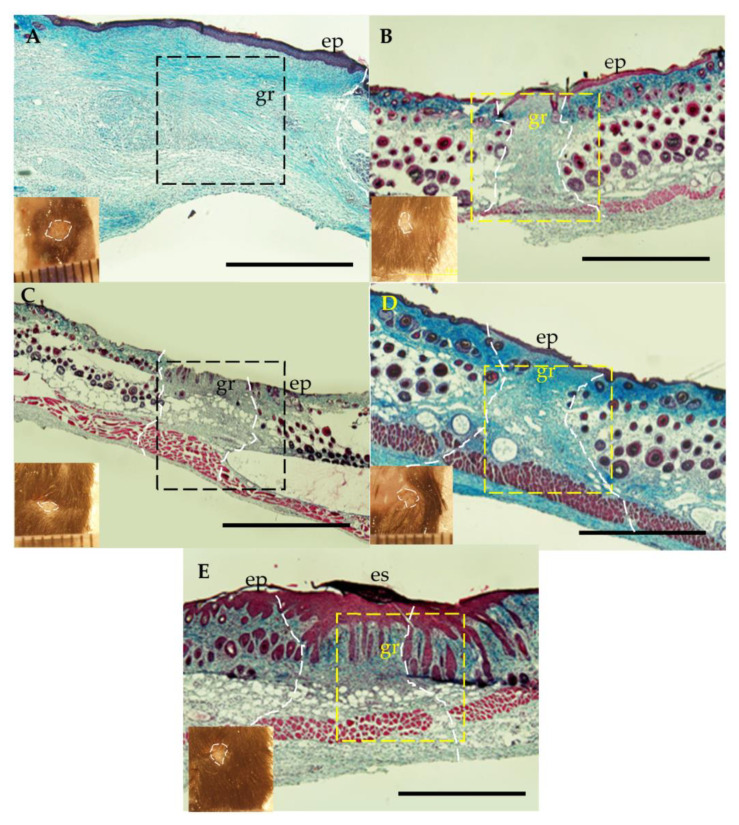
Representative trichrome histological image of the harvested tissue on day 14 after treatment with control (**A**), PEG-D4-Si (**B**), PEG-D6-Si (**C**), PEG-D8-Si (**D**), and PEG-D6 (**E**). Black (**A**,**C**) and yellow (**B**,**D**,**E**) dash lines indicate the area used to calculate collagen content. White dash line indicates the wound proximities. es = eschar, gr = granulation tissue, and ep = epidermis. Scale bars are 1 mm.

**Figure 9:**
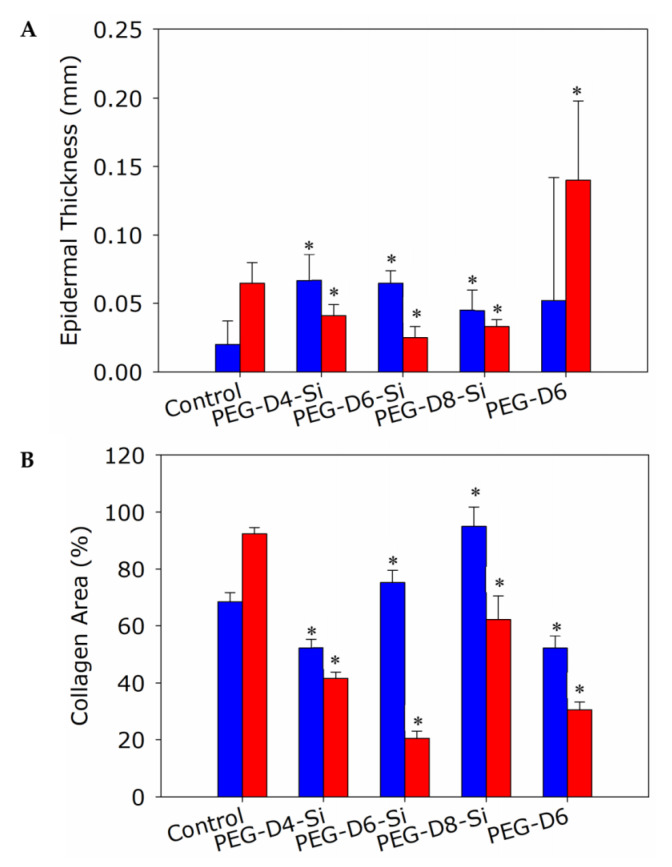
The epidermis thickness (**A**) and collagen content (**B**) of the wound treated with various adhesive formulations at days 7 (■) and 14 (■) post-surgery. Number of repeats is 3. * *p* < 0.05 when compared to the control at the given time point.

Masson’s trichrome histological staining of the wounds was used to evaluate the wound morphologies, determine the epidermal thicknesses, and determine the collagen contents (Figure 7 and Figure 8). From the images captured on day 7, the wounds appeared to be irregular in shape, resulting from dermal contractions [38]. Among the SiP-containing adhesives, the PEG-D4-Si-treated wounds exhibited the most prominent level of granulation tissue. This observation may be attributed to the elevated level of H_2_O_2_ released by the PEG-D4-Si (~80 µM), when compared to those released by the PEG-D6-Si and PEG-D8-Si. Additionally, the adhesive-treated wounds exhibited a thicker epidermis when compared to the untreated control (Figure 9A). This dermal hyperplasia, or the thickening of the epidermis, is likely due to the application of H_2_O_2_ to the wound site [10,39]. Among the composite adhesives, PEG-D4-Si released the highest amount of H_2_O_2_ and resulted in the thickest epidermis layer that was measured. The PEG-D6-treated wound also exhibited a thicker epidermis, but this increase was not significantly different from the other adhesive-treated wounds. This indicated that the increase in the thickness of the regenerated epidermis was not only affected by the level of H_2_O_2_, but was also affected by the SiP, which can release soluble silica. Additionally, an accumulation of hyaluronic acid at the wound site could be attributed to the observed thickening of the epidermis during the early phase of wound healing [40]. Hyaluronic acid promotes keratinocytes activation and the proliferation that is necessary for rapid re-epithelialization [41].

By day 14, the tissue samples exhibited wound remodeling for all the adhesive-treated samples. There was a reduction in the wound size and an increase in the granulation tissue for all the adhesive formulations (Figure 8). The wounds treated with the composite adhesives all demonstrated a reduction in their collagen content (Figure 9B), with the PEG-D6-Si-treated wounds demonstrating the lowest collagen content. This suggests that the wound was remodeled successfully by day 14, with reduced granulation tissue [32,42]. Most importantly, treatment with an SiP-containing adhesive resulted in a reduction in the epidermal thickness, with PEG-D6-Si demonstrating the thinnest epidermal layer of around 25 µm. The epidermal thicknesses in these treatment groups were similar to those of healthy mice (~20 µm) [43]. This indicates that treatment with an SiP-containing adhesive regenerates new tissues, closely resembling those of a healthy epidermis. On the other hand, both the untreated control and the treatment with the SiP-free PEG-D6 resulted in an increase in the epidermal thickness, indicating prolonged epidermal hyperplasia.

**Figure 10:**
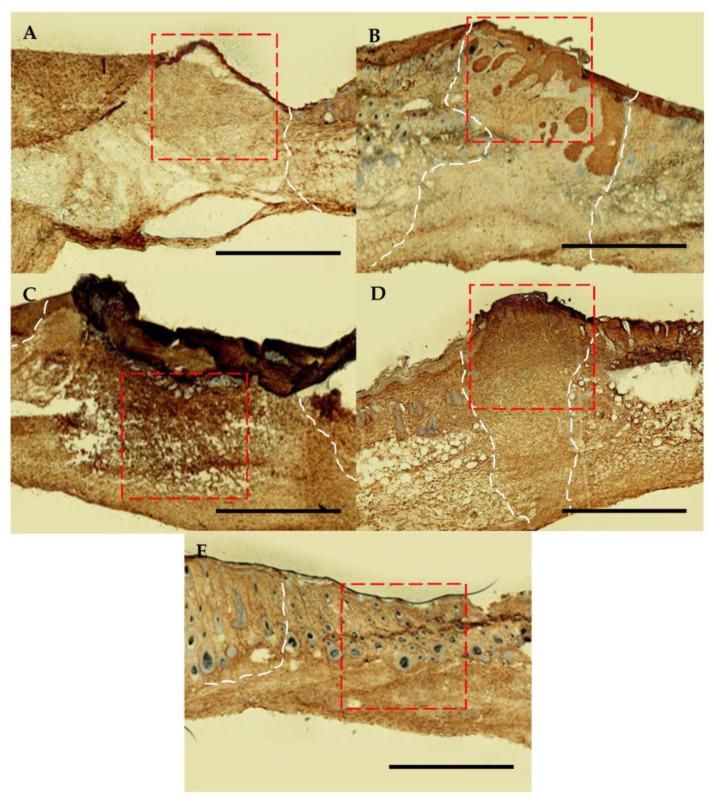
Representative keratin-6 histological staining images of the harvested tissue at day 7 of control (**A**), PEG-D4-Si (**B**), PEG-D6-Si (**C**), PEG-D8-Si (**D**), and PEG-D6 (**E**). Red dash line indicates the area used to calculate keratinocyte content. White dash line indicates the wound proximities. Scale bars are 1 cm.

**Figure 11:**
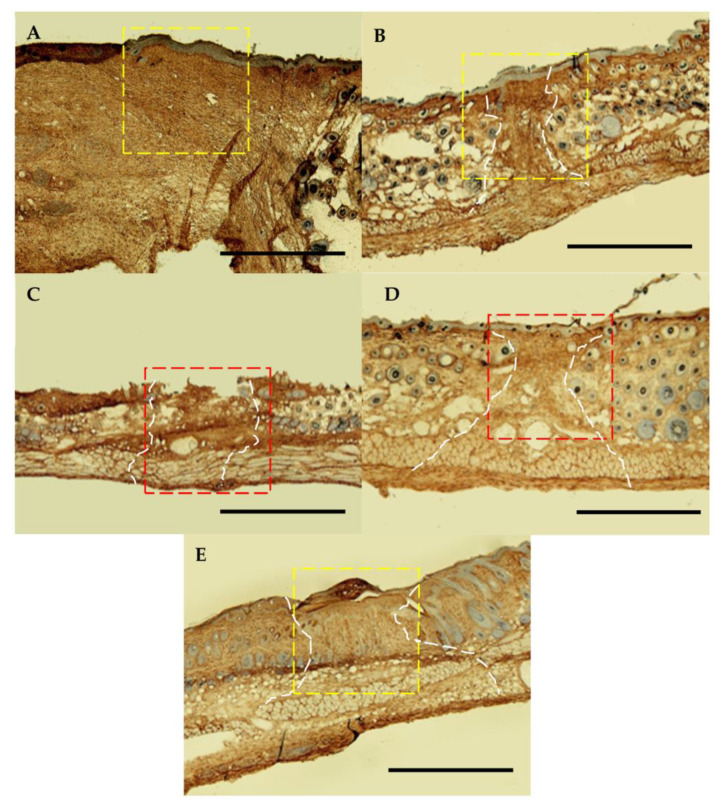
Representative keratin-6 histological staining images of the harvested tissue at day 14 of control (**A**), PEG-D4-Si (**B**), PEG-D6-Si (**C**), PEG-D8-Si (**D**), and PEG-D6 (**E**). Red and yellow dash lines indicate the area used to calculate keratinocyte content. White dash line indicates the wound proximities. Scale bars are 1 cm.

**Figure 12:**
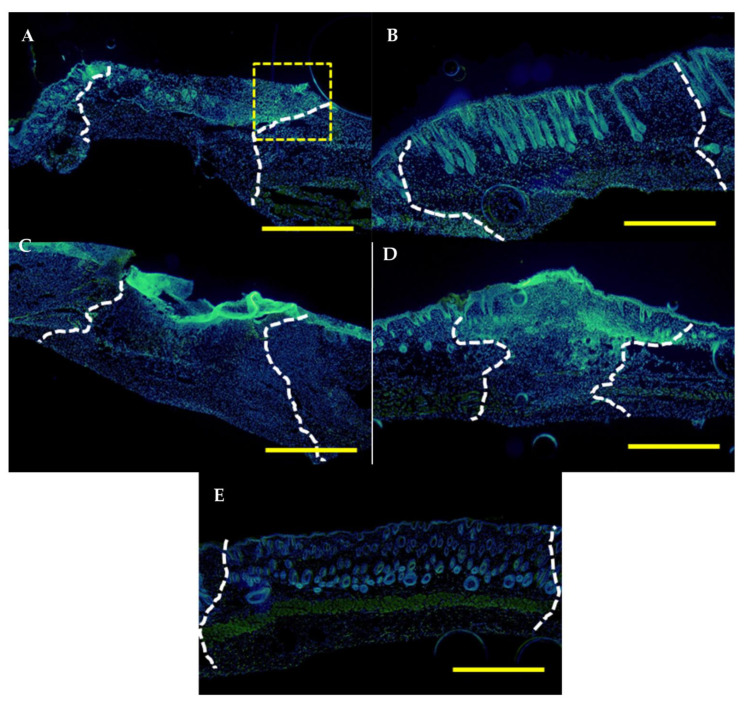
Representative CD68-DAPI immunological image of the harvested tissue on day 7 after treatment with control (**A**), PEG-D4-Si (**B**), PEG-D6-Si (**C**), PEG-D8-Si (**D**), and PEG-D6 (**E**). Yellow dash line box gives the example for the area used to calculate CD68-positive cells. White dash line indicates the wound proximities. Green = CD68-positive cells and blue = cell nucleus. Scale bars are 1 mm.

**Figure 13:**
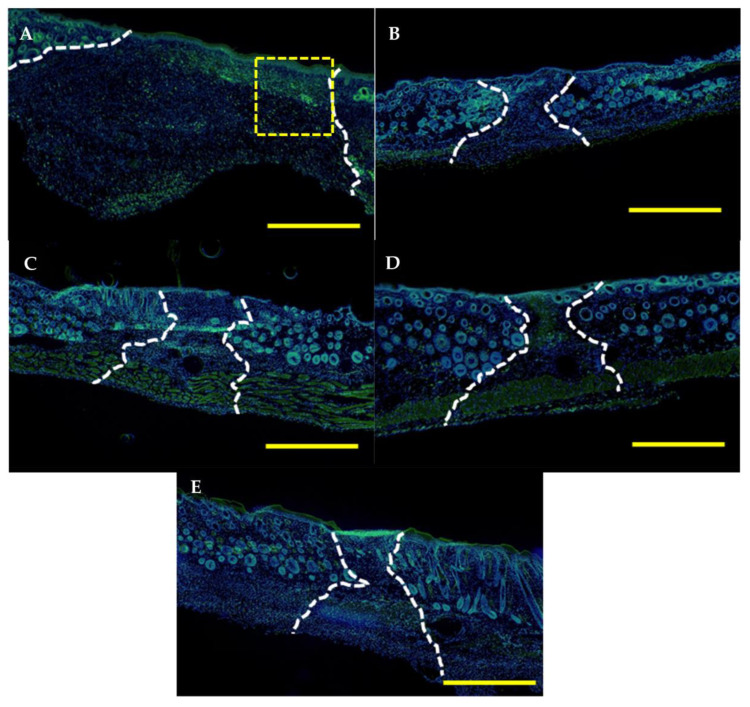
Representative CD68-DAPI immunological image of the harvested tissue on day 14 after treatment with control (**A**), PEG-D4-Si (**B**), PEG-D6-Si (**C**), PEG-D8-Si (**D**), and PEG-D6 (**E**). Yellow dash line box gives the example for the area used to calculate CD68-positive cells. White dash line indicates the wound proximities. Green = CD68-positive cells and blue = cell nucleus. Scale bars are 1 mm.

**Figure 14:**
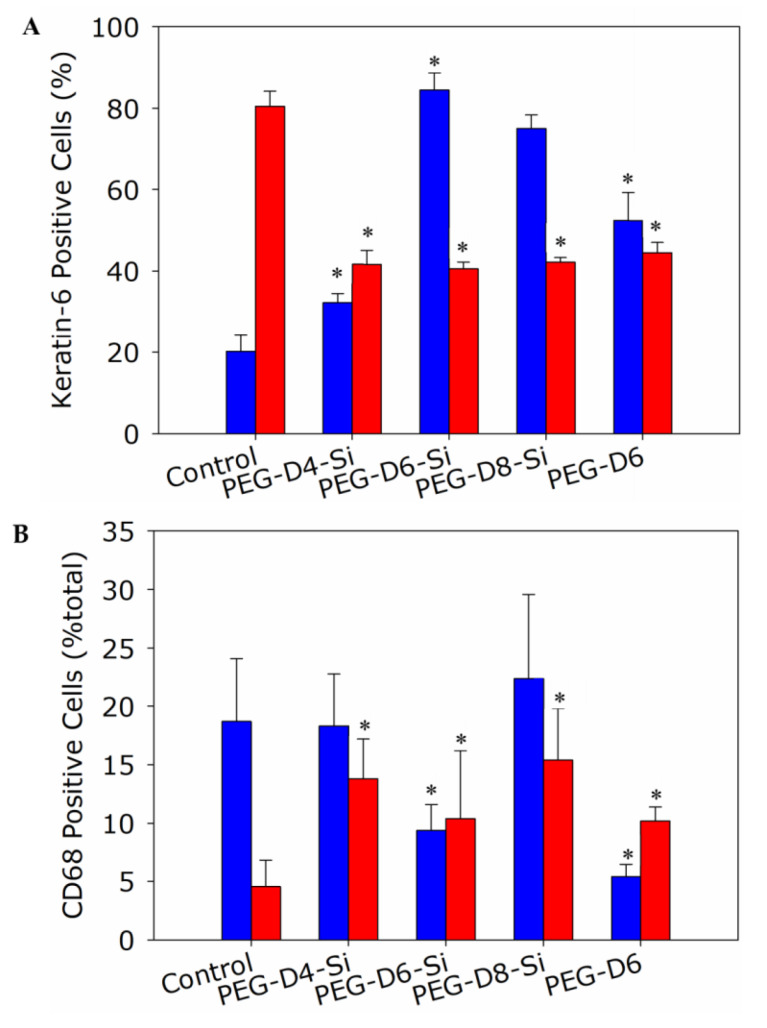
Percentage of keratin-6- (**A**) and CD68- (**B**) positive cells in the wound site treated with various adhesive formulations on days 7 (■) and 14 (■) post-surgery. Number of repeats is 3. * *p* < 0.05 when compared to the control at the given time point.

The wounds were further stained with keratin-6 (Figure 10 and Figure 11) and CD68 (Figure 12 and Figure 13) to evaluate the presence of keratinocytes and macrophages at the wound site. On day 7, the percentage of the keratin-6-positive cells in the untreated controls was significantly lower (~20%) when compared to the adhesive-treated wounds (Figure 14A). The release of H_2_O_2_ from the adhesive likely recruited immune cells and served as a chemotaxis source for recruiting the keratinocytes. For PEG-D6-Si and PEG-D8-Si, the percentages of the keratin-6-positive cells were around 80%. This indicated that treatment with an adhesive increased the keratinocyte recruitment to the wound site at an early time point. The presence of keratinocytes indicated the maturation of the skin. The maturation of keratinocytes leads to skin cornification, which provides a protective outer layer for the underlying dermal tissue [44]. Additionally, these keratinocytes were concentrated in the epidermis and its surroundings, resembling the structure of healthy skin tissue [42,45]. The released H_2_O_2_ likely recruited the keratinocytes to the wound site and promoted its proliferation as a response to oxidative stress [10,37]. Similarly, soluble silica has previously been demonstrated to induce keratinocyte migration and proliferation [25,46]. By day 14, the controls exhibited elevated keratin-6-positive cells compared to the adhesive-treated wounds. For the wounds treated with the composite adhesives, although the average keratin-6-positive cells in the area surveyed was around 40%, this value was over 80% near the epidermis. This indicates that the adhesive treatment promoted an early keratinocyte recruitment and the maturation of the healed wound [47,48].

In the untreated control, the percentage of the CD68-positive cells was found to be at around 18% on day 7, which was later reduced to around 4% by day 14 (Figure 14B). On day 7, both the PEG-D6- and PEG-D6-Si-treated wounds exhibited significantly lower CD68-positive cells when compared to the control, indicating a reduced macrophage recruitment. Conversely, on day 14, the percentages of the CD68-positive cells for all the adhesive-treated wounds were significantly higher than that of the control. Although this increase in the macrophage population may suggest a prolonged immune response, CD68 does not distinguish between the types of macrophages that are present. There are two types of macrophages: M1 macrophages, which are involved in inflammatory response, and M2 macrophages, which are involved in matrix remodeling, the suppression of inflammatory responses, and tissue regeneration [5]. Although additional work may be required to distinguish these two macrophage types, the combined results of the reduced collagen content and the regeneration of the epidermal thickness to similar to that of healthy tissues suggest that the inflammatory response ceased by day 14 for the composite adhesive-treated wounds.

Taken together, a catechol-containing bioadhesive that generates H_2_O_2_ as a by-product, in combination with SiP, can be used to promote dermal wound healing without resulting in epidermal hyperplasia. Specifically, PEG-D6-Si was found to be the ideal formulation for accelerating this dermal wound healing. PEG-D6-Si demonstrated a combination of fast wound closure and the regeneration of the thin epidermis that is found in healthy skin. PEG-D6-Si released around 60 µM of H_2_O_2_, which is consistent with previous findings that have indicated that this level of H_2_O_2_ is desirable for promoting wound healing [10,37,49]. In addition to H_2_O_2_, the incorporation of SiP also contributed to promoting this wound healing, as the wounds that were treated with the SiP-free PEG-D6 resulted in epidermal hyperplasia. The incorporated SiP can release soluble silica, which has been demonstrated to induce keratinocyte and fibroblast proliferation and migration, as well as epidermal formation and tissue remodeling [42,44,46]. The dermal wound healing was performed by using composite adhesives with the same composition (i.e., the same PEG and SiP concentrations), which releases varying amounts of H_2_O_2_ (40–80 µM) while maintaining the performance of the adhesive. This experiment was uniquely designed to investigate the effect of the H_2_O_2_ concentration on wound healing, while minimizing the contributions from other parameters (e.g., the effect of the composition). The adhesive and filler combination that is reported here could potentially be further tuned to tailor a H_2_O_2_ release profile that may be more suited for the repair of other tissues. Specifically, the adhesive formulation could be chosen based on the predicted values from the results of the robust design experiment.

## 4. Conclusions

The ability of a composite adhesive consisting of PEG-modified catechol and SiP to heal full-thickness dermal wounds was evaluated. Given the large number of factors and factor levels, robust design was utilized to select the adhesive formulations that released the suitable amounts of H_2_O_2_ for wound healing. Although the prediction from the robust design experiment was generally poor for the gelation times and adhesion strengths, the predicted and experimental values for the H_2_O_2_ concentrations were in good agreement. The chosen adhesive formulations possessed the same compositions and mechanical properties, with the only varying parameter being the different amount of H_2_O_2_ concentrations generated by each formulation. This experimental design enabled us to study the effect of H_2_O_2_ concentration on dermal wound healing, while minimizing the contributions of the other factors. From the dermal wound healing experiment on mice, all the adhesive-treated wounds increased the rate of wound closure when compared to the untreated control. Additionally, the composite adhesive promoted dermal wound healing without resulting in epidermal hyperplasia. The release of H_2_O_2_ from the catechol and soluble silica from the SiP contributed to recruiting keratinocytes to the wound site in order to effectively promote the wound healing. As a result, PEG-D6-Si is the optimal formulation for accelerating wound closure, wound remodeling, and the maturation of a skin wound.

## Figures and Tables

**Table 1:** Factor and factor levels utilized for the robust design experiment.

		Factor			
		A	B	C	D
		PEG Architecture	PEG Conc. (mg/mL)	PBS Conc.	wt% SiP
**Factor Level**	**1**	4-arm	75	0.5**×**	0
	**2**	6-arm	113	1**×**	5
	**3**	8-arm	150	2**×**	10

**Table 2:** Nine adhesive formulations tested to fulfill the robust design matrix requirement.

Formulations	Factor			
	PEG Architecture	PEG Conc. (mg/mL)	PBS Conc.	wt% SiP
1	4-arm	75	0.5**×**	0
2	4-arm	113	1**×**	5
3	4-arm	150	2**×**	10
4	6-arm	75	1**×**	10
5	6-arm	113	2**×**	0
6	6-arm	150	0.5**×**	5
7	8-arm	75	2**×**	5
8	8-arm	113	0.56**×**	10
9	8-arm	150	1**×**	0

**Table 3:** Percent relative variation of each factor on the performance of the adhesive.

Factor	% Relative Variation		
	Gelation Time	Adhesive Strength	H_2_O_2_ Concentration
PEG Architecture	1.1%	3.4%	65.6%
PEG Concentration	78.6%	93.8%	8.2%
PBS Concentration	2.1%	2.4%	5.5%
SiP wt%	18.2%	0.47%	20.7%

## Data Availability

The data are available from the corresponding author upon reasonable request.

## Supplementary Materials

The following supporting information can be downloaded at: https://www.mdpi.com/article/10.3390/polym15081905/s1, Methods used for data analysis associated with robust design experiment, results from the robust design experiment, ^1^H NMR spectra, formulation assignment for the animal study, and exemplary tissue sections of histological staining; Figure S1: Chemical structures of PEG-D4 (A), PEG-D6 (B), PEG-D8 (C), and each arm of the branched PEG containing glutaric ester and terminal dopamine group (D); Figure S2: ^1^H NMR spectrum of 4-arm (A), 6-arm (B), and 8-arm (C) PEG-DA, and associated peak assignments (D); Figure S3: Representative scanning transmission electron microscopy images of porous SiP; Figure S4: Schematic representation of the dermal wound healing model in mice (A). Representative photographs of the dermal wounds (B), dermal wounds enclosed within a medical-grade silicon ring (C), a dermal wound treated with an adhesive (D), dermal wounds covered by a non-adhering dressing (Adaptic®) (E), and then a breathable adhesive film (Hydrofilm®) (F); Figure S5: Average signal-to-noise ratios for gelation time (A), adhesive strength (B), and max H_2_O_2_ concentration (C) based on the 9 adhesive formulations from Table S2. Values are plotted as mean and standard deviation of *η* values for the corresponding factor level; Table S1: Experimental results of the nine formulations used in the robust design experiment; Table S2: Predicted adhesive performance for PEG-D4; Table S3: Predicted adhesive performance for PEG-D6; Table S4: Predicted adhesive performance for PEG-D8; Table S5: Adhesive formulations chosen for dermal wound repair based on their predicted adhesive performance; Table S6: Control and treatment groups tested in the full thickness dermal wound model. References [28,29] are cited in the Supplementary Materials.
