# Supplementary material for: Utilizing Robust Design to Optimize Composite Bioadhesive for Promoting Dermal Wound Repair
Source: Polymers (Basel). 2023 Apr 15;15(8):1905. doi: 10.3390/polym15081905 (PMC10144490; doi:10.3390/polym15081905)
Supplement: Supplementary file 1 [file polymers-15-01905-s001.zip › polymers-2252304-supplementary.pdf]

Supplementary Materials

Utilizing Robust Design to Optimize Composite Bioadhesives for Promoting Dermal Wound Repair

Rattapol Pinnaratip [1], Zhongtian Zhang [1], Ariana Smies [1], Pegah Kord Forooshani [1], Xiaoqing Tang [2] Rupak M. Rajachar [1,3] and Bruce P. Lee [1,*]

[1] Department of Biomedical Engineering, Michigan Technological University, Houghton, MI 49931, USA; rpinnara@mtu.edu (R.P.)

[2] Department of Biological Sciences, Life Science and Technology Institute, Michigan Technological University, Houghton, MI 49931, USA

[3] Marine Ecology and Telemetry Research (MarEcoTel), Seabeck, WA 98380, USA

* Correspondence: bplee@mtu.edu

Data Analysis Associated with the Robust Design Experiment:

A robust design experiment was employed to determine the relative degree to which particular factors (the PEG architecture, PEG concentration, PBS concentration, and SiP concentration) affected the performance of the composite adhesive [1,2] The average values of the test results (e.g., the gelation times, lap shear adhesion strengths, and H_2O_2 concentrations) of nine adhesive formulations (**Table S2**) were utilized to determine the signal-to-nose ratio (S/N) for each formulation using **Equation (S1)**:

$$\mathrm{S/N} = -10 \times \log\left(\frac{\left(\sum_{i=1}^{n}\left(1/Y_i^2\right)\right)}{n}\right) \tag{S1}$$

where Y_i is the individual experimental value and n is the number of repeats for the experiment. These nine η values were used to calculate the % relative variation or the relative contributions of each factor to the measured outcomes, which is defined by **Equation (S2)**:

$$\%\ \text{relative variation} = \frac{SS_{factor}}{SS_{total}} \times 100 \tag{S2}$$

where SS_{factor} and SS_{total} are the sum of squares due to a factor and the total sum of squares, respectively. They are found by **Equations (S3)** and (**S4)**, respectively.

$$SS_{factor} = 3(m_x - m)^2 + 3\left(m_y - m\right)^2 + 3(m_z - m)^2 \tag{S3}$$

where m_x, m_y, and m_z are each the mean of the three η values from the experiments performed at each factor level, and m is the grand mean of the nine η values.

$$SS_{total} = \sum_{i=1}^{9}(\eta_i - m)^2 \tag{S4}$$

where η_1 through η_9 are the nine η values determined for the nine adhesive formulations.

To make a prediction of the adhesive performance, the predicted signal-to-nose ratio (η_{pred}) was first calculated using **Equation (S5)**:

$$\eta_{pred} = m + (m_{Ai} - m) + (m_{Bi} - m) + (m_{Ci} - m) + (m_{Di} - m) \tag{S5}$$

where m_{Ai}, m_{Bi}, m_{Ci}, and m_{Di} are the mean η values for the corresponding factors (i.e., A = PEG architecture, B =PEG concentration, C = PBS concentration, and D = SiP

concentration) and the subscript *i* corresponds to the factor level (i.e., $i = 1, 2,$ or 3). The predicted value (y_{pred}) was found using **Equation (S6)**:

$$y_{pred} = \sqrt{10^{-\frac{\eta_{pred}}{10}}} \tag{S6}$$

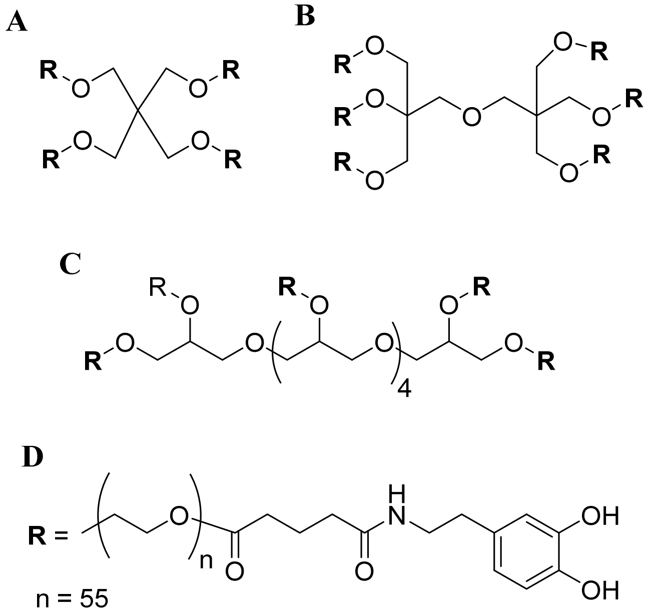

Figure S1. Chemical structures of PEG-D4 (A), PEG-D6 (B), PEG-D8 (C), and each arm of the branched PEG containing glutaric ester and terminal dopamine group (D).

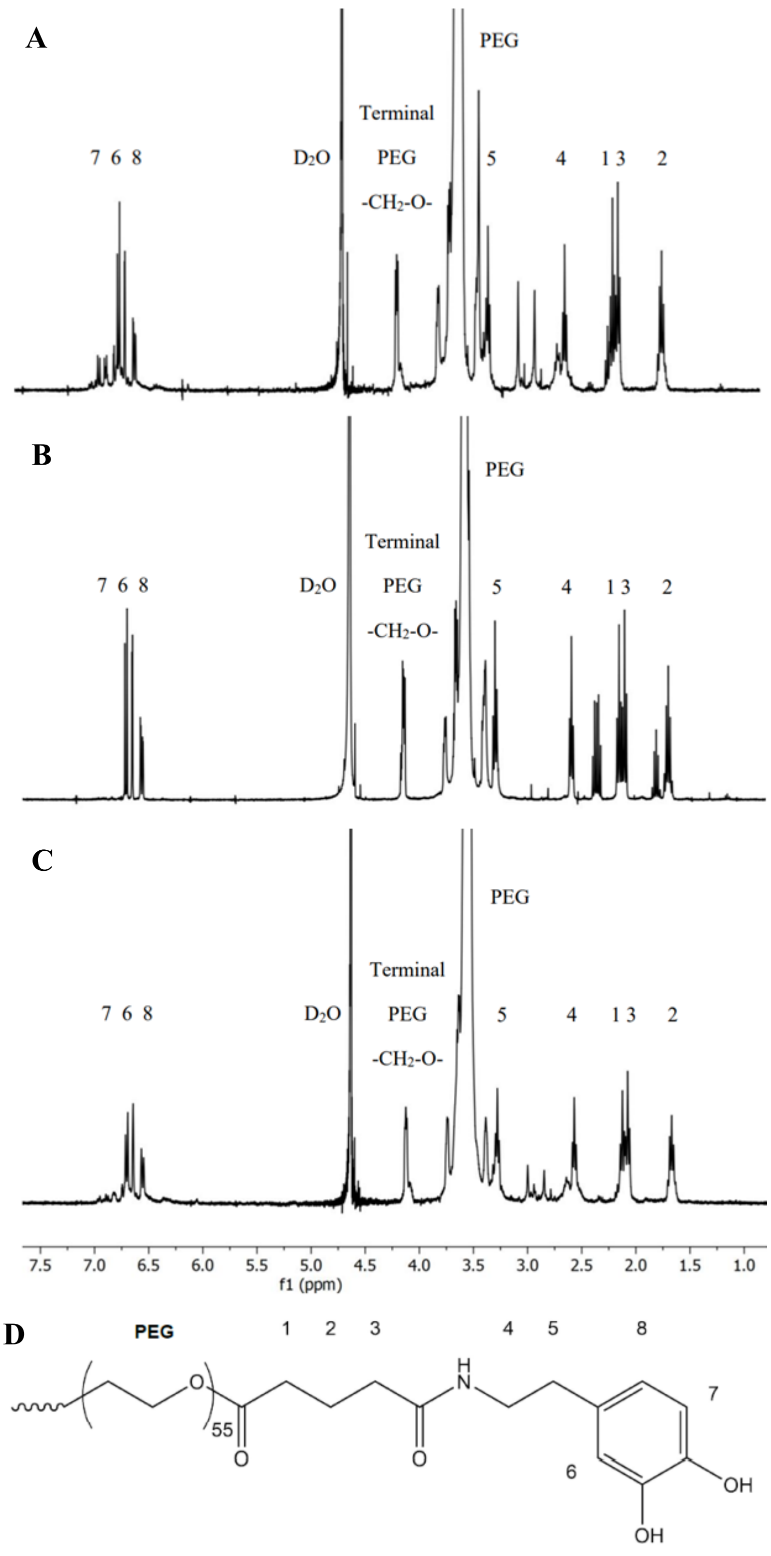

Figure S2. ^{1}H NMR spectrum of 4-arm (A), 6-arm (B), and 8-arm (C) PEG-DA, and associated peak assignments (D).

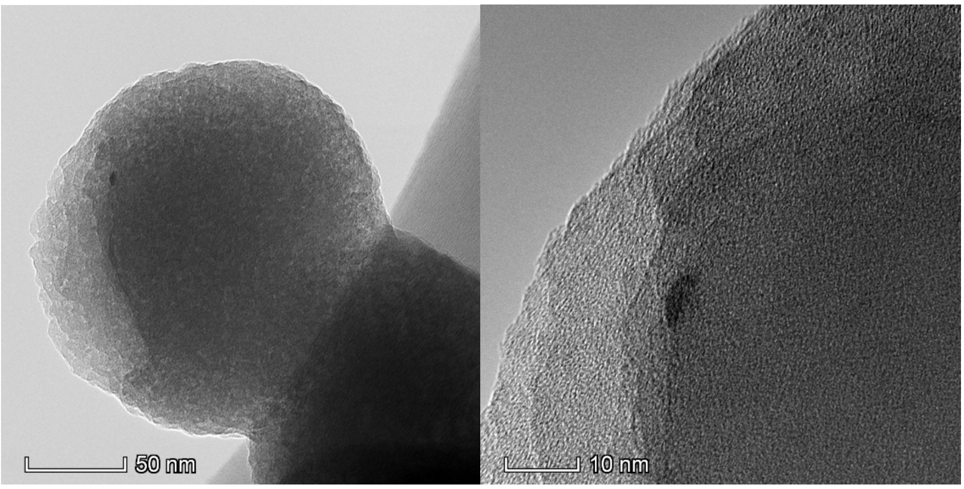

Figure S3. Representative scanning transmission electron microscopy images of porous SiP.

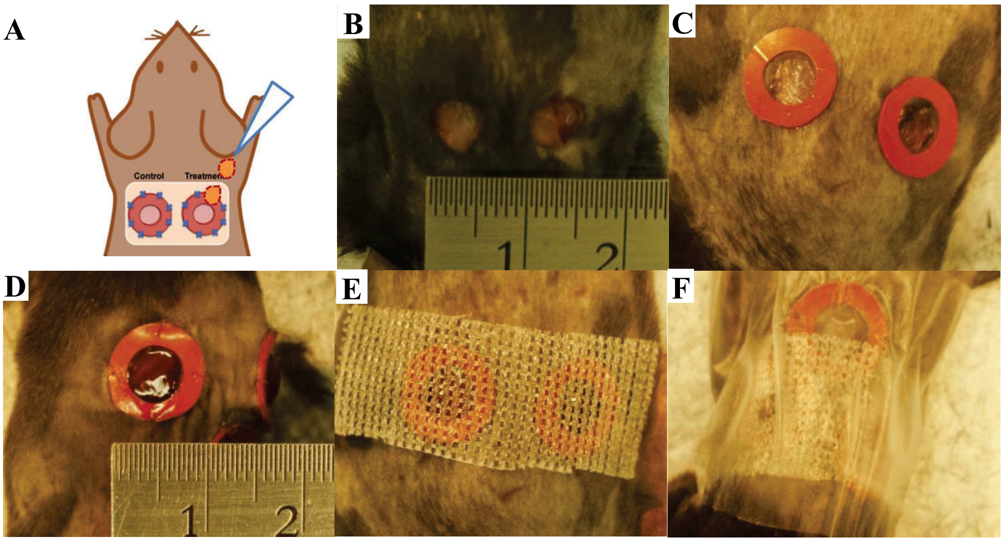

Figure S4. Schematic representation of the dermal wound healing model in mice (A). Representative photographs of the dermal wounds (B), dermal wounds enclosed within a medical-grade silicon ring (C), a dermal wound treated with an adhesive (D), dermal wounds covered by a non-adhering dressing (Adaptic®) (E), and a breathable adhesive film (Hydrofilm®) (F).

Table S1. Experimental results of the nine formulations used in the robust design experiment.

Formulation	Gelation Time (second)	Adhesive Strength (kPa)	Max H_2O_2 Conc. (μM)
1	273.33 ± 8.98	N/A	92.35 ± 2.01
2	45.83 ± 3.44	3.17 ± 0.18	89.30 ± 1.72
3	167.50 ± 4.79	3.62 ± 0.29	68.31 ± 2.14
4	515.00 ± 13.84	N/A	52.36 ± 1.91
5	39.17 ± 1.68	3.10 ± 0.20	78.17 ± 2.08
6	52.83 ± 2.27	5.31 ± 0.16	72.22 ± 2.15
7	358.33 ± 7.45	N/A	26.85 ± 2.59
8	85.50 ± 4.23	4.26 ± 0.29	32.33 ± 1.49
9	37.50 ± 4.79	7.07 ± 0.63	61.35 ± 3.01

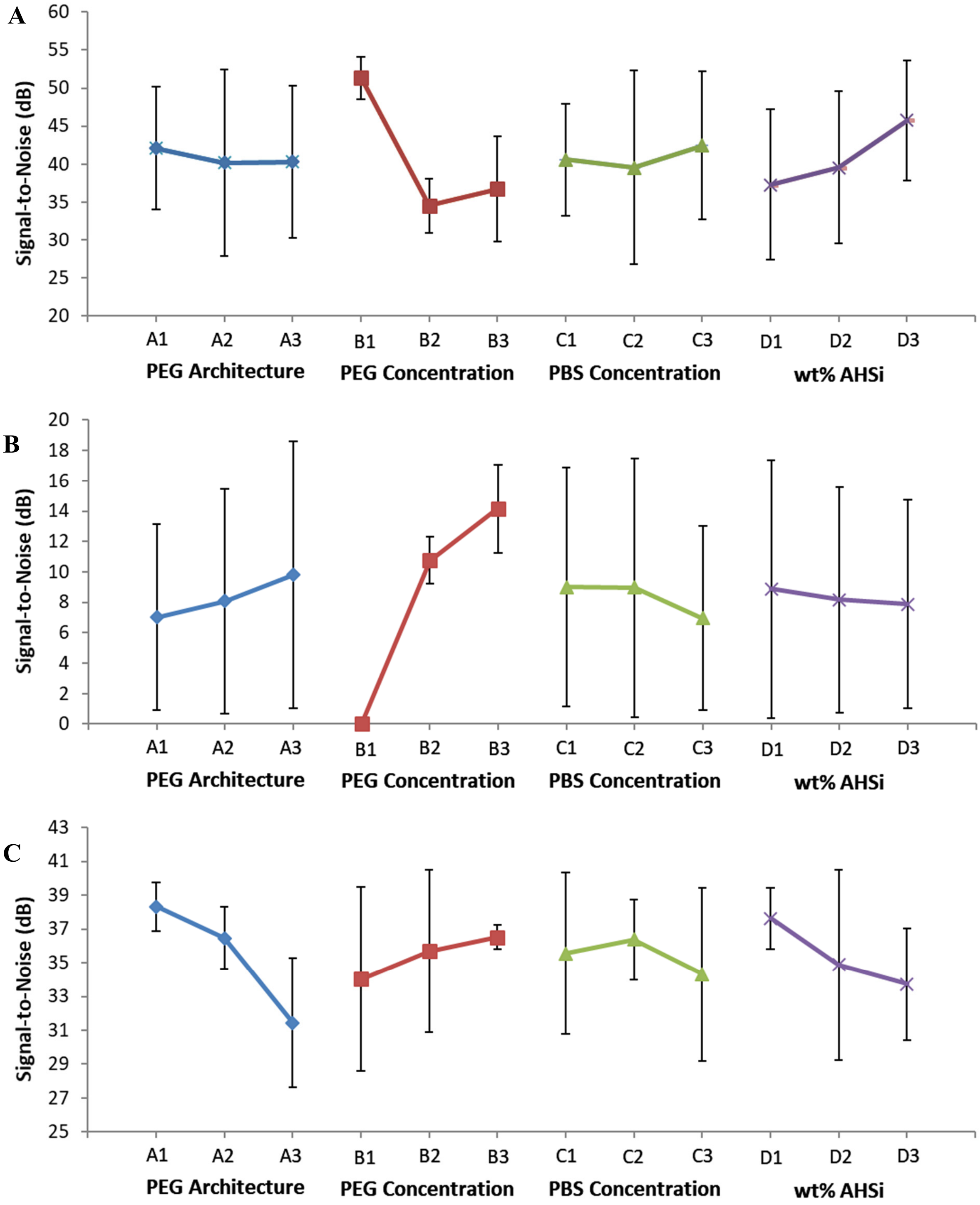

Figure S5. Average signal-to-noise ratios for gelation times (A), adhesive strengths (B), and max H_2O_2 concentrations (C) based on the 9 adhesive formulations from **Table S2**. Values are plotted as means and standard deviations of η values for the corresponding factor levels.

Table S2. Predicted adhesive performance for PEG-D4.

Factors			Predicted Values		
**PEG Conc. (mg/mL)**	**PBS Conc.**	**SiP wt%**	**Gelation Time (second)**	**Adhesive Strength (kPa)**	**H_2O_2 Conc. (μM)**
75	0.5×	0	272.89	1.00	92.29
		5	354.88	0.92	67.23
		10	725.72	0.89	58.96
	1×	0	242.06	0.99	101.45
		5	314.78	0.91	73.91
		10	643.73	0.88	64.82
	2×	0	338.63	0.79	80.21
		5	440.38	0.73	58.43
		10	900.56	0.70	51.25
113	0.5×	0	39.39	3.45	111.45
		5	51.22	3.17	81.19
		10	104.75	3.07	71.21
	1×	0	34.94	3.43	122.51
		5	45.44	3.15	89.25
		10	92.91	3.05	78.28
	2×	0	48.88	2.73	96.86
		5	63.56	2.50	70.57
		10	129.98	2.43	61.89
150	0.5×	0	50.69	5.11	122.82
		5	65.92	4.69	89.48
		10	134.82	4.54	78.47
	1×	0	44.97	5.08	135.02
		5	58.48	4.67	98.36
		10	119.58	4.52	86.27
	2×	0	62.91	4.03	106.75
		5	81.81	3.71	77.77
		10	167.29	3.59	68.21

Table S3. Predicted adhesive performance for PEG-D6.

Factors			Predicted Values		
PEG Conc. (mg/mL)	PBS Conc.	SiP wt%	Gelation Time (second)	Adhesive Strength (kPa)	H_2O_2 Conc. (μM)
75	0.5×	0	218.09	1.13	74.39
		5	283.61	1.04	54.20
		10	579.98	1.01	47.53
	1×	0	193.45	1.12	81.78
		5	251.57	1.03	59.58
		10	514.45	1.00	52.25
	2×	0	270.62	0.89	64.66
		5	351.94	0.82	47.10
		10	719.71	0.79	41.31
113	0.5×	0	31.48	3.90	89.84
		5	40.94	3.59	65.45
		10	83.71	3.47	57.40
	1×	0	27.92	3.88	98.76
		5	36.31	3.56	71.95
		10	74.25	3.45	63.10
	2×	0	39.06	3.08	78.08
		5	50.80	2.83	56.88
		10	103.88	2.74	49.89
150	0.5×	0	40.51	5.77	99.01
		5	52.69	5.30	72.13
		10	107.74	5.14	63.26
	1×	0	35.94	5.74	108.84
		5	46.73	5.27	79.29
		10	95.57	5.11	69.54
	2×	0	50.27	4.55	86.05
		5	65.38	4.19	62.69
		10	133.70	4.05	54.98

Table S4. Predicted adhesive performance for PEG-D8.

Factors			Predicted Values		
**PEG Conc. (mg/mL)**	**PBS Conc.**	**SiP wt%**	**Gelation Time (second)**	**Adhesive Strength (kPa)**	**H_2O_2 Conc. (μM)**
75	0.5×	0	221.91	1.38	41.77
		5	288.58	1.27	30.43
		10	590.14	1.23	26.69
	1×	0	196.83	1.37	45.92
		5	255.97	1.26	33.45
		10	523.46	1.22	29.34
	2×	0	275.37	1.09	36.30
		5	358.10	1.00	26.45
		10	732.31	0.97	23.20
113	0.5×	0	32.03	4.76	50.44
		5	41.65	4.37	36.75
		10	85.18	4.23	32.23
	1×	0	28.41	4.73	55.45
		5	36.95	4.35	40.40
		10	75.56	4.21	35.43
	2×	0	39.75	3.76	43.84
		5	51.69	3.45	31.94
		10	105.70	3.34	28.01
150	0.5×	0	41.22	7.04	55.59
		5	53.61	6.47	40.50
		10	109.63	6.26	35.52
	1×	0	36.57	6.99	61.11
		5	47.55	6.43	44.52
		10	97.24	6.23	39.04
	2×	0	51.15	5.55	48.32
		5	66.52	5.11	35.20
		10	136.04	4.94	30.87

Table S5. Adhesive formulations chosen for dermal wound repair based on their predicted adhesive performances.

Formulation Name	Factor				Predicted Values		
	PEG Architecture	PEG Conc. (mg/mL)	PBS Conc.	SiP wt%	Gelation Time (second)	Adhesive Strength (kPa)	H_2O_2 Conc. (μM)
PEG-D4-Si	4	150	1×	10	119.58	4.52	86.27
PEG-D6-Si	6	150	1×	10	95.57	5.11	69.54
PEG-D8-Si	8	150	1×	10	97.24	6.23	39.04
PEG-D6	6	150	1×	0	35.94	5.74	108.84

Table S6. Control and treatment groups tested in the full-thickness dermal wound model

Treatment Groups	Note
Control	Left untreated
PEG-D6	6-arm PEG with no SiP
PEG-D4-Si	4-arm PEG containing 10 wt% SiP
PEG-D6-Si	6-arm PEG containing 10 wt% SiP
PEG-D8-Si	8-arm PEG containing 10 wt% SiP

References

1. Phadke, M.S. *Quality Engineering Using Robust Design*; Prentice Hall: Englewood Cliffs, NJ, USA, 1989; p. 334.
2. Roy, R.K. *Design of Experiments Using the Taguchi Approach: 16 Steps to Product and Process Improvement*; John Wiley & Sons: New York, NY, USA, 2001.
